# A service evaluation of the multidisciplinary team approach to hypodontia

**DOI:** 10.1038/s41415-023-6385-5

**Published:** 2023-10-13

**Authors:** Faye Doughty, Sruthi Pillai, Dylan Hamill, Nadine Amin, Martin P. Ashley

**Affiliations:** 41415449468001 Dental Core Trainee and Academic Clinical Fellow in Oral Surgery, University Dental Hospital of Manchester, Manchester University NHS Foundation Trust, M15 6FH, UK; 41415449468002 Dental Core Trainee, University Dental Hospital of Manchester, Manchester University NHS Foundation Trust, M15 6FH, UK; 41415449468003 Consultant in Restorative Dentistry, University Dental Hospital of Manchester, Manchester University NHS Foundation Trust, M15 6FH, UK

## Abstract

**Introduction**Patients with hypodontia can be seen by a multidisciplinary team clinic (MDT) for treatment planning at the University Dental Hospital of Manchester (UDHM). The MDT consists of orthodontics, restorative dentistry and oral surgery colleagues.

**Aims and methods**A retrospective case-note analysis was conducted on 558 hypodontia patients seen on Manchester Hypodontia Clinic (MHC) between 2016-2022 to assess service utilisation and treatment planning outcomes.

**Results**The average age of patients attending the MHC was 16 (range 8-50). The distribution of mild, moderate and severe hypodontia in the sample was 28%, 37% and 35%, respectively. Most common treatments proposed were fixed appliances, extractions, implants and resin-bonded bridges. Out of 558 patients seen for consultation on the MHC, 365 (65%) were accepted for treatment. The average number of visits for treatment was 15.5 (range: 1-55). The average number of did not attend/was not brought appointments, patient cancellations and hospital cancellations were 0.8, 1.4 and 1.8, respectively.

**Conclusion**Hypodontia patients referred to UDHM are triaged by consultants in orthodontics or restorative dentistry, and if MDT planning is required, they are booked onto the MHC. There are sufficient patients with complex cases of moderate and severe hypodontia to justify a regular MDT hypodontia clinic.

## Key points

- Hypodontia is a complex condition that can be effectively managed by a specialist, multidisciplinary team.
- There are sufficient patients with moderate and severe hypodontia to justify a regular multidisciplinary regional clinic. Most patients with moderate and severe hypodontia will require orthodontics, oral surgery and restorative dentistry treatment to achieve a good outcome.
- Many patients with mild hypodontia either don't access dental care at all or may be effectively screened in primary care and by assessment with a specialist orthodontist or restorative dentist and do not need to be referred to the hypodontia MDT.

## Introduction

Hypodontia is defined as the developmental absence of one or more teeth, with the exception of third molars,^1^ with a prevalence of 6-7% depending on the population studied. Hypodontia can be classified according to the number of missing teeth:^2^

- Mild = 1-2
- Moderate = 3-5
- Severe = 6 or more.

Hypodontia is a significant dental condition that often leads to functional and aesthetic concerns for affected patients and their families. It usually requires complex and lengthy specialist dental treatment to achieve an acceptable outcome.^2^

In the UK, it has been recognised for almost 30 years that a multidisciplinary approach for managing patients with hypodontia is appropriate.^3^ Since then, a large number of multidisciplinary teams (MDT) have developed around the UK and several publications related to treatment approaches for these patients have been published.^2^^,^^4^^,^^5^^,^^6^^,^^7^^,^^8^

In many centres, before the development of a dedicated hypodontia MDT clinic, provision of treatment for these patients would have been poorly co-ordinated, and the clinical decision-making was based on the preferences of individual clinicians. Opportunities for treatment and clinical outcomes would have varied considerably, depending on access to local and regional hypodontia MDT services. Collaboration between specialist colleagues within local MDTs and between MDTs across the UK, such as within the Restorative Dentistry UK (RD-UK) national hypodontia clinical excellence network (CEN), has reduced variation in service design and clinical pathways and has led to improved patient experience^9^ and clinical outcomes.

**Fig. 1:**
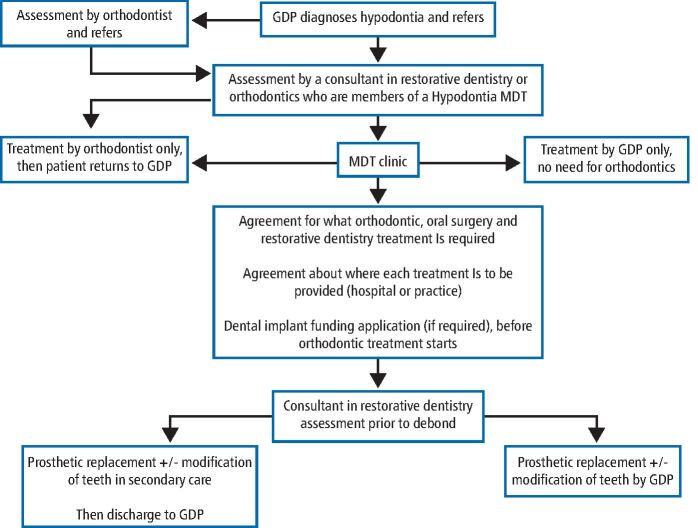
A local hypodontia care pathway used at UDHM, illustrating the stages that most hypodontia patients will follow from initial attendance and diagnosis with their GDP, and if referred, through each decision-making and treatment stage, to discharge. This pathway is comparable to all others presented within the RD-UK hypodontia CEN

Patients are usually diagnosed with hypodontia in late childhood or early teenage years by their general dental practitioner (GDP) when some of their permanent teeth fail to erupt as expected.^10^A referral to an appropriately qualified and experienced colleague is the first of a series of well-established stages within the hypodontia care pathway (Fig. 1). As well as continuing to receive ongoing dental care from their GDP, the patient will follow a number of stages through the hypodontia care pathway, depending on their clinical condition and desire to have treatment.

In 2010, the Manchester Hypodontia Clinic (MHC) was developed to co-ordinate and improve the quality of treatment provided for patients with hypodontia. Information gathered at each clinic led to improved service efficiencies and patient experiences achieved by the MDT approach.^9^

Further data collection at the monthly MHC has continued since then and the move from paper case notes to an electronic patient record system in July 2016 has enabled greater access to and analysis of information related to patient treatment pathways and outcomes.

Members of the MHC MDT include two consultant orthodontists, two consultants in restorative dentistry and one consultant in oral surgery. Speciality registrars from each discipline and other postgraduate and undergraduate students also regularly attend MHC. It is noted that some UK hypodontia MDTs include a consultant in paediatric dentistry and although the MHC does not include one as a core member, they are located in an adjacent clinic and attend whenever required.

## Methods

Between January 2010 and December 2022, 1,925 patients attended the MHC MDT. Comparison of the initial service efficiency and patient experience outcomes reported in 2013^9^ with those up to December 2022 confirm the same high-quality care outcomes continue to be achieved (Table 1).

**Table 1:** Patient experience and service efficiency outcomes achieved at the MHC. Analysis of 1,925 patients attending between January 2010 and December 2022

Did the staff introduce themselves?	100%
Did you receive a clear explanation of why you are attending the clinic?	99%
Do you feel you were involved enough in your appointment?	98%
Do you know what has been decided about your treatment?	98%
Was it worthwhile attending the clinic?	99%
Patients rating their experience of attending the clinic	69% excellent, 30% good
Patients seen within 10 minutes of their appointment time (MHC standard)	56%
Patients seen within 30 minutes of their appointment time (Trust standard)	99%
Average length of time at the clinic	21 minutes

The MHC was specifically included in the Care Quality Commission report related to the 2018 hospital inspection, in which the University Dental Hospital of Manchester (UDHM) was rated as 'outstanding'.^11^

A further retrospective analysis of electronic patient records for all patients who attended the MHC between July 2016 and December 2022 has been conducted. It was not possible to access all of the same data elements from previous, paper-based records. Several different parameters related to patient demographics, service utilisation and clinical pathways were collected and analysed (Table 2). Data were collected by four clinicians. The first ten entries were collected as a group for familiarisation and standardisation of the process and for calibration purposes.

**Table 2:** Parameters for which data was collected and analysed for patients attending the MHC from July 2016 to December 2022

Demographics	Service utilisation	Clinical outcomes
Age at first appointment Sex Source of referral Severity of hypodontia	Number of DNA/WNB to appointments Number of patient cancellations Number of hospital cancellations Number of appointments after treatment is completed	Speciality input Patient outcome (accepted for treatment vs sent back to referrer) Proposed treatment plan Changes to treatment plans Restoration with fixed appliances, RBBs or implants Total number of visits Complications

## Results

Between July 2016 and December 2022, 945 patients were booked to attend the MHC. Paediatric patients (aged below 16) were classed as 'was not brought' (WNB) if they failed to attend their appointment, and adult patients aged 16 and over who failed to attend their appointment were recorded as 'did not attend' (DNA). Due to patient DNA/WNB and cancellations, 839 patients (88.8%) attended the MHC and data were subsequently collected on these patients. This is comparable with all other consultation clinic attendance data at UDHM. All those who cancelled were offered a further appointment on the next MHC. In keeping with local policy, all those who DNA/WNB to their appointment were contacted and offered a further appointment if requested.

Out of the 839 patients seen on the MDT clinic, 558 patients had hypodontia. Patients without hypodontia were also seen at the clinic so they could benefit from an MDT treatment plan due to the complexity of their cases. These cases include traumatic loss of teeth, root resorption causing early loss of teeth, and impacted teeth. For the purpose of this paper, all non-hypodontia patient data have been removed. Only the 558 hypodontia cases will be included and discussed.

The mean age of the hypodontia patients who attended the MHC was 16.8 years, with a range of 8-50 years old. The average was skewed by the outliers who were older adults referred to the clinic. The mode age was 14 and the median age was 15. This age range of attendance at the MHC is in keeping with the patient's age when hypodontia is first suspected by the GDP.^10^ A female predominance was observed: of the patients who attended, 56% (n = 315) were female and 44% (n = 243) were male.

All hypodontia patients are referred to the UDHM by either their GDP, a specialist practitioner in primary care or from another secondary care hospital service. Patients are then triaged by a consultant and if appropriate for the MHC, they are added to the MDT clinic. The referral criteria are simply that the patient has a diagnosis of hypodontia or another dental developmental anomaly and may benefit from MDT planning. For those cases where an MDT approach is deemed unnecessary, an appropriate treatment plan is described and shared with the referring clinician. The most common source of internal referral was orthodontics (n = 412; 74%), followed by restorative dentistry (n = 91; 16%) and paediatric dentistry (n = 28; 5%). A small number of patients (n = 24; 4.3%) were referred directly to the MHC from primary care and other hospitals, in situations that supported a fast-track approach.

The severity of hypodontia for those attending the MDT was relatively even and is outlined in Table 3.

**Table 3:** The severity of hypodontia affecting the patients attending the MHC

Severity of hypodontia	Number of patients (%)
Mild (1-2 congenitally missing teeth)	158 (28%)
Moderate (3-5 congenitally missing teeth)	206 (37%)
Severe (6+ congenitally missing teeth)	194 (35%)

Of the 558 hypodontia patients seen on the MHC between July 2016 and December 2022, 365 (65%) were accepted for treatment at UDHM and 193 (35%) were sent back to the referrer with a detailed treatment plan formulated by the MDT.

For those hypodontia patients who attended the MHC and then subsequently attended UDHM for a course of treatment, the mean number of total appointments attended was 15.5 (range 1-55). The mean number of DNA/WNBs during a course of treatment was 0.8 (range 0-10). Patient cancellations had a mean of 1.4 (range 0-14) and hospital cancellations had a mean of 1.8 (range 0-17).

The mean number of appointments attended after treatment was completed was 0.6 (range 0-14). This was due to a number of reasons, including lost retainers, planned reviews and also due to complications.

A treatment plan was proposed for all hypodontia patients attending the clinic (n = 558). Proposed treatments have been summarised in Table 4.

**Table 4:** Proposed treatments for 558 hypodontia patients who attended the MHC between July 2016 and December 2022

Treatment proposed	Number of patients (%)
Monitor, no treatment proposed	17 (3%)
**Orthodontic treatment**	
Fixed appliances	463 (83%)
Removable appliances	91 (16.3%)
**Oral surgery treatment**	
Extractions	250 (44.8%)
Exposure of tooth ± bonding of gold chain	42 (7.5%)
Cyst decompression/marsupialisation	1 (0.2%)
Frenectomy	6 (1.1%)
Dental implants	157 (28.1%)
**Restorative dentistry treatment**	
RBB	157 (28.1%)
Direct restorations	105 (18.8%)
Crowns and veneers	6 (1.1%)
Dentures	32 (5.7%)
Endodontic treatment	10 (1.8%)
Periodontal treatment	4 (0.7%)
RBB/denture/dental implants (to be decided later)	123 (22%)
**Oral and maxillofacial surgery treatment**	
Orthognathic surgery	13 (2.3%)

Most patients required a combination of various treatments, often including all dental specialties represented in the MDT, validating the clinic design to ensure the patient's treatment plan was discussed and agreed by all those involved.

The most common treatments proposed were fixed orthodontic appliances (n = 463; 83%) and tooth extractions (n = 250; 44.8%). Tooth replacement with bridges and dental implants and tooth modification with composite restorations were each planned for around one-fifth of cases. In 123 cases, prosthetic replacement to restore gaps was yet to be decided. This decision was to be made later in the patient's treatment, around or after the orthodontic debond appointment. However, at the initial appointment, all options are discussed with the patient and parent/guardian. Where possible, the creation of suitable three-dimensional spaces through orthodontic treatment is completed, to allow the full range of restorative dentistry treatment options to be provided, including partial dentures, bridges and dental implants.

**Fig. 2:**
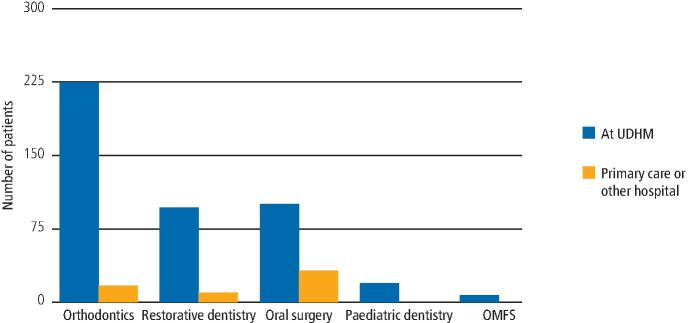
The type of speciality dental treatment required and where it was delivered

Out of the 365 patients accepted for treatment at UDHM between July 2016 and December 2022, 123 have completed treatment and 242 are currently either on treatment waiting lists or are progressing through active treatment. Speciality treatment input is illustrated in Figure 2. Some patients were seen in primary care or in another hospital for some aspects of their orthodontics, restorative dentistry and oral surgery treatments. They were then seen for other treatment components at UDHM. For example, some simple tooth extractions were carried out in general dental practice and the patient was then seen for the rest of their treatment at UDHM.

Most hypodontia patients who had completed treatment required treatment from multiple specialties (n = 77; 63%). Out of the 123 completed hypodontia cases, 37% (n = 46) required treatment from one speciality, 42% required treatment from two specialties (n = 51) and 21% (n = 26) required treatment from three specialties.

Table 5 outlines how many fixed appliances, resin-bonded bridges (RBBs) and dental implants have been carried out in those accepted for treatment at UDHM (365 patients). In some cases, part of the treatment was carried out in primary care or at another hospital and the rest was carried out at UDHM. Fixed appliances were provided by UDHM in 221 cases (60.5%) and in a different hospital/primary care in 16 cases (4.4%). RBBs were provided by UDHM in 36 cases (9.9%) and in a different hospital/primary care in five cases (1.4%). Implants were provided by UDHM in 21 cases (5.8%) and in a different hospital/primary care in two cases (0.5%). There is a discrepancy between the number of planned and completed fixed appliances, RBBs and dental implants. This is due to the fact that some patients will still be waiting to commence orthodontic treatment and some are in active orthodontic treatment, and so are yet to reach the restorative phase of treatment.

**Table 5:** Number of hypodontia patients accepted for treatment at UDHM (n = 365) who received fixed appliances/RBBs/implants

	Number of hypodontia patients			
Most common treatment provided	Mild	Moderate	Severe	Total
Fixed appliances completed at UDHM	67	85	69	221
Fixed appliances completed in primary care or a different hospital	0	10	6	16
RBB completed at UDHM	11	15	10	36
RBB completed in primary care or a different hospital	2	1	2	5
Implant completed at UDHM	2	10	9	21
Implant completed in primary care or a different hospital	1	0	1	2

Changes to the proposed treatment plan were noted in 11.6% of cases, which is to be expected with such complex cases. Out of the 13 planned cases for orthognathic surgery, two patients changed their minds and opted for non-surgical treatment.

Only seven (1.9%) patient complications were noted in the 365 hypodontia patients accepted for treatment at UDHM. Complications included fixed appliances not tolerated by the patient, RBB replacement as the patient was unhappy with the shade, RBB de-bond, orthodontic relapse, implant failure and subsequent replacement, and loss of tooth vitality due to dental trauma.

## Discussion

The MHC is a well-established, regional MDT clinic, with consistently high patient experience and service efficiency outcomes.^9^ Having access to demographic and clinical data for such a large cohort of patients is unusual and is of value for service design, service improvement and clinical standards comparisons. As the clinicians contribute to UK CENs, it is useful to assess this MDT model alongside those used elsewhere.

Analysis of the demographic data confirms the age and sex of those who access the service is as expected: generally, teenagers and young adults, but with older adults also requiring specialist dental care.

The dental assessment of the growing child by the GDP identifies the lack of expected tooth eruption and as is appropriate, most patients are initially referred for an orthodontic opinion. A total of 75% of patients who then attended the MHC did so having previously been referred to and seen in a hospital orthodontic clinic.

The proportion of patients with mild (n = 158; 28%), moderate (n = 206; 37%) or severe (n = 194; 35%) hypodontia seen on the MHC is not typical for the population in general. The hypodontia pattern observed in the general population is mild (81.6%), moderate (14.3%) and severe (3.1%).^12^ The majority of patients seen on the hypodontia clinic had either moderate or severe hypodontia (missing three or more teeth).

There are a number of potential reasons for this difference. A significant number of patients do not access primary dental care, either by personal choice or due to lack of availability of clinical services in a convenient location for them. Of those that do attend, ideally the GDP will be vigilant for unusual tooth eruption patterns and if necessary, offer referral to a colleague more experienced in managing hypodontia patients. The patients with less complex presentations will either be reassured or offered treatment locally by these colleagues, rather than being referred to a regional hypodontia MDT. At UDHM, consultants in either orthodontics or restorative dentistry triage all hypodontia referrals, and if they feel mild hypodontia cases can be effectively managed without an MDT approach, they will instead be booked onto the relevant single speciality clinic (such as orthodontics or restorative dentistry). Conversely, the difference may also reflect inadequate standards of clinical practice, with either missed diagnoses, poor understanding of local referral pathways, or unequal access to specialist dental services. Therefore, patients with mild hypodontia in a general hypodontia population (81%) compared to those that have mild hypodontia that attend the MHC (28%) probably both demonstrates the effectiveness of screening and assessment by many GDPs in primary care and specialists in secondary care and also, possibly some aspects of inadequate practice in both settings. There will inevitably be many patients affected by hypodontia of all patterns who do not get to benefit from the hypodontia care pathway approach to screening, treatment planning and delivery.

Despite the development of hypodontia care pathways, including the MDT approach to treatment planning and delivery, it is clear that a hypodontia patient must successfully navigate several stages of the pathway (access to a GDP, diagnosis, referral to a specialist, treatment planning or referral to a regional MDT) if treatment is to be planned and delivered to what is expected to be a higher standard. This is complex and requires successful contribution of the patient and many colleagues.

Service utilisation between 2016 and 2022 for patients attending the MHC and subsequently receiving treatment was similar to UDHM-wide attendance and cancellation figures over the same time period. These figures were undoubtedly impacted by the necessary restrictions to service provision during the COVID-19 pandemic. The time from the start of treatment to completion has been prolonged and fewer patients were able to start a course of treatment, leading to longer delays at each stage of the hypodontia care pathway. This has especially affected and reduced the delivery of elective oral surgery procedures under general anaesthetic, such as either extraction or exposure and bonding of impacted teeth.

The majority of hypodontia patients required input from two or more dental specialties during their treatment (n = 77; 63%). This justifies the multidisciplinary approach to treatment planning for patients with moderate to severe hypodontia adopted on this clinic. Although a consultant in paediatric dentistry is not routinely present at the MHC, one is always locally available to give an opinion when required. Some patients are internally referred from the paediatric dentistry department and a proportion of patients require some treatment within paediatric dentistry too (3.6%; n = 20).

Outcomes reported in this paper are relevant to the UK population where patients receive healthcare within the NHS, a state-funded system. All proposed treatments can be provided across multiple specialties within a hospital setting, utilising secondary care specialists and primary care clinicians in local dental practices. Dental implants can be funded by the NHS under specific circumstances outlined by local NHS commissioning groups in keeping with national guidelines.^13^ This includes patients with congenitally missing teeth if conventional treatments such as adhesive bridges and dentures are inappropriate. Uptake of treatment plans and clinical outcomes may have differed if patients had to fund either some or all of their treatment.

Research into quality of life of hypodontia patients identifies that the condition impacts upon emotional and social wellbeing.^14^ This highlights the importance of oral rehabilitation in these patients, to restore both aesthetics and function. However, to achieve these outcomes requires a multidisciplinary approach and significant commitment from the patient and their families, often over an extended period of time. A multidisciplinary approach is utilised to ensure thorough treatment planning and improved outcomes for patients.

## Conclusion

Hypodontia is a complex condition that can be effectively managed by a specialist MDT. There are sufficient patients with moderate and severe hypodontia to justify a regular multidisciplinary regional clinic.

Patients referred to UDHM with mild hypodontia are effectively screened by consultants and only attend the MHC if an MDT approach is deemed to be necessary. Mild cases not requiring MDT care are booked onto the appropriate single speciality clinics, such as orthodontics or restorative dentistry. Guidance for GDPs is offered in another paper in this themed series.^10^ Most patients with moderate to severe hypodontia will require orthodontics, restorative dentistry and/or oral surgery treatment to achieve a good outcome. These patients are therefore referred onto the MHC for treatment planning.

Although this paper reports on the MHC, the existence of several other regional hypodontia MDTs across the UK presents an opportunity for close collaboration towards development of clinical guidelines, service improvement and clinical research, based on collection of a minimum dataset and delivery of patient care through unified care pathways.
